# Using beta-binomial regression for high-precision differential methylation analysis in multifactor whole-genome bisulfite sequencing experiments

**DOI:** 10.1186/1471-2105-15-215

**Published:** 2014-06-24

**Authors:** Egor Dolzhenko, Andrew D Smith

**Affiliations:** 1 Molecular and Computational Biology Section, Division of Biological Sciences, University of Southern California, Los Angeles, California, USA

**Keywords:** Epigenomics, Differential methylation, Beta-binomial regression

## Abstract

**Background:**

Whole-genome bisulfite sequencing currently provides the highest-precision view of the epigenome, with quantitative information about populations of cells down to single nucleotide resolution. Several studies have demonstrated the value of this precision: meaningful features that correlate strongly with biological functions can be found associated with only a few CpG sites. Understanding the role of DNA methylation, and more broadly the role of DNA accessibility, requires that methylation differences between populations of cells are identified with extreme precision and in complex experimental designs.

**Results:**

In this work we investigated the use of beta-binomial regression as a general approach for modeling whole-genome bisulfite data to identify differentially methylated sites and genomic intervals.

**Conclusions:**

The regression-based analysis can handle medium- and large-scale experiments where it becomes critical to accurately model variation in methylation levels between replicates and account for influence of various experimental factors like cell types or batch effects.

## Background

DNA methylation is a chemical modification of DNA resulting from the addition of a methyl group to a DNA nucleotide. In vertebrates, DNA methylation – which chiefly occurs at cytosines within CpG dinucleotides – has been associated with numerous biological functions. For example, methylation plays a key role in genomic imprinting, X-chromosome inactivation [1,2], and has been associated with suppression of transposable elements during embryonic development [3]. Numerous studies have shown correlation between promoter methylation and gene expression. Furthermore, the presence of large-scale abnormally methylated genomic regions is a hallmark feature of many types of cancers [4].

Whole-genome bisulfite sequencing (WGBS) is currently the state-of-the-art technology for obtaining a comprehensive, nucleotide-resolution view of the epigenome. A key step in WGBS is the bisulfite treatment of DNA designed to convert unmethylated cytosines to uracils and leave the methylated cytosines unchanged. During sequencing, unmethylated cytosines are read out as thymines. In this way, the presence of mismatches in the aligned reads can determine the methylation states of the cytosines in the DNA molecules that gave rise to the reads. Typical WGBS experiments involve DNA molecules originating from many distinct cells and, consequently, the methylation state of a particular cytosine may differ from one molecule to another. Because of this, methylation of a single cytosine in the context of WGBS experiments is described by its *methylation level* or the fraction of molecules in the sample where that cytosine is methylated (see also [5]). Therefore, methylation levels can be estimated from the proportions of reads indicating methylation at each site.

The epigenetic differences between groups of replicate samples are typically described by individual differentially methylated (DM) sites (e.g. individual cytosines or CpG dinucleotides) and DM regions – regions dominated by DM sites. Detection of methylation changes between groups of replicates requires taking into account variation of methylation levels within each group. Such variation could be attributed to a variety of technical and biological sources including different library preparation protocols, unequal cytosine conversion rates, or the natural epigenetic variation between individuals [6]. For example, Rakyan and others [7] highlighted some distributions of methylation levels across replicates that could arise in the context of epigenome-wide association studies.

A number of approaches currently exist for assessing differential methylation from WGBS data. One of the most straightforward and commonly used methods for comparing epigenomes of a pair of samples is Fisher’s Exact Test [8-11]. There are also DM detection algorithms based on hidden Markov models (HMMs). A recently released tool ComMet, included in the Bisulfighter methylation analysis suite [12], is also designed to detect DM regions and DM sites between two samples. Another HMM-based DM detection method is included in the MethPipe methylation analysis pipeline [13,14]. This method first uses HMMs to detect lowly methylated regions, called hypo methylated regions (HMRs) for each sample and then constructs DM regions from the fragments of HMRs. Existing methods based on Fisher’s Exact Test and HMMs are appropriate for comparing a pair of samples at a time (coming either directly from the experiment or obtained by pooling other samples); however, they lack the ability to account for variability of methylation levels between replicates.

Another variety of DM detection algorithms are based on smoothing. These methods operate under the assumption that methylation levels vary smoothly along the genome. They use local smoothing to estimate the true methylation level of each site in each sample. For example, the DM detection algorithm included in the BSmooth methylation analysis pipeline [15] is designed to compute DM regions between two groups of samples. After smoothing, BSmooth performs a statistical test, similar to the t-test, to find DM sites which form DM regions. BiSeq [16] is another method based on smoothing. Unlike BSmooth, it can be used for experiments that go beyond comparing two groups of samples, but it requires a set of candidate regions that may exhibit differential methylation. Thus BiSeq is suitable for the analysis of data from reduced representation bisulfite sequencing (RRBS) and other experiments designed to assess methylation of a specific set of genomic intervals. Because smoothing-based methods perform smoothing on each sample individually, care must be taken when dealing with regions where methylation levels are difficult or impossible to estimate due to very low or no coverage, and regions where methylation has sharp changes (e.g. transcription factor binding sites). This said, smoothing-based methods have been demonstrated to facilitate accurate and reproducible differential methylation analysis [15].

A few recently released DM-detection methods are based on the beta-binomial distribution. The beta-binomial, which has first been used for modeling WGBS proportions by Molaro and others [17], is a natural choice for describing methylation levels of an individual site across replicates as it can account for both sampling and epigenetic variability. A method implemented in the bioconductor package DSS [18] constructs a genome-wide prior distribution for the beta-binomial dispersion parameter and then uses it to estimate the distribution of methylation levels in each group of replicates. The differentially methylated sites are determined by testing the means of these distributions for equality. The MOABS algorithm [19] constructs a genome-wide distribution of methylation levels and then uses it to estimate the distribution of methylation levels at individual sites. The significance of differential methylation is subsequently determined by an estimate of the methylation difference between the two groups of replicate samples. The precision with which these methods determine if a given site is differentially methylated depends on how closely does the distribution of site’s methylation levels across replicates or the dispersion parameter resembles the genome-wide prior.

Another category of DM detection algorithms are based on regression. BiSeq, mentioned earlier, performs a beta regression after smoothing and so also fits into this category. MethylKit [20] uses logistic regression to test for differential methylation at individual sites; its model assumes that the number of reads indicating methylation follows a binomial distribution across replicates.

The existing methods for detecting differential methylation lack either the ability to analyze WGBS datasets in complex experimental designs or the ability to account for variation across biological replicates. These limitations reduce the usefulness of current methods for analysis of multifactor WGBS datasets that are emerging in the contexts of epigenome-wide association studies (EWAS) and other studies aiming to answer questions about groups and populations of individuals. Here we introduce a novel DM-detection method based on beta-binomial regression that overcomes these limitations.

## Methods

We start by discussing the utility of the beta-binomial regression for modeling methylation levels of individual sites (e.g. C, CpGs, CHH, CHG) across multiple samples. This approach is especially useful in the context of epigenome-wide association studies (EWAS) that typically involve methylomes of many individuals and, potentially, numerous sites with complicated methylation profiles across the replicates.

As mentioned in the introduction, the methylation level of an individual site is the fraction of molecules in the sample that have a methyl group at that site. This level can be computed separately for each strand, but we will assume throughout that the methylation level refers to both strands. Assume that *n* reads from the WGBS experiment map over a given cytosine, and that the cytosine is methylated in *m* of these reads. Then (*m*,*n*) is the read proportion corresponding to the site. In the absence of any biological or technical variation, *m*∼binom(*p*,*n*), where *p* is the unknown methylation level of the site. So the unbiased estimator for *p* is $\hat{p}=m/n$.

However, it is widely recognized that variation exists and arises from multiple biological and technical sources [7,15]. Thus, when dealing with multiple replicates, we must associate some uncertainty with each methylation level. Let *p*_*i*_ denote the methylation level of the site in the *i*th replicate. (This way, *p*_*i*_s give the methylation level of the cytosine under consideration across all available replicates). The typical assumption is *p*_*i*_∼Beta(*α*,*β*) for some shape parameters *α*≥0 and *β*≥0. Using the beta distribution in such analysis, however, requires that we know the values of *p*_*i*_ as the basis for inferences about the *α* and *β*. If we use ${\hat{p}}_{i}$s directly, we are ignoring any uncertainty in their estimates. While this is appropriate for studies based on BeadArray technology [21], which estimate each *p*_*i*_ based on interrogating very large numbers of molecules, there are many important and emerging contexts in which sequencing-based studies will involve low values of *n*_*i*_ (coverage of the cytosine in sample *i*).

The coverage issue discussed in the previous paragraph can be addressed by using the beta-binomial distribution instead of the beta. The beta-binomial distribution retains the flexibility of beta in modeling the distribution of methylation levels across replicates and, at the same time, takes into account the uncertainty associated with coverage.

### Beta-binomial distribution

The beta-binomial is a compound distribution obtained from the binomial by assuming that its probability of success parameter follows a beta distribution. The beta-binomial is obtained from Binom(*p*,*n*) by assuming *p*∼Beta(*α*,*β*) resulting in the probability mass function

(1) Pr(M=m|n,α,β)=nmB(m+α,n−m+β)B(α,β),

where *B* is the beta function. Reparametrization *π*:=*α*/(*α*+*β*) and *γ*:=1/(*α*+*β*+1) yields

E(M)=nπandVar(M)=nπ(1−π)(1+(n−1)γ).

The parameter *π* is the analog of the binomial probability of success parameter which can be interpreted as the average methylation level of a set of replicate samples. The parameter *γ* is called the *dispersion parameter*. Observe that the binomial distribution is a special case of beta-binomial distribution with *γ* equal to 0.

### Beta-binomial regression

We use beta-binomial regression [22] to individually model the methylation levels of every site across the given set of samples. Let *M*_1_,…,*M*_*s*_ be the i.i.d random variables corresponding to the number of reads indicating methylation of the site in the samples 1,…,*s* so that

$$M_{i}∼\text{BetaBinomial}\left(n_{i},\pi_{i},\gamma \right).$$

We assume that $\pi_{i}=g\left(\overset{t}{\underset{j=1}{\sum }}X_{i,j}\eta_{j}\right)$, where *g* is a link function, *X* is an *s*×*t* model matrix, and *η* is a *t*×1 vector of regression parameters. Finally, *γ* is a common dispersion parameter. The columns of the model matrix correspond to the binary experimental factors (e.g. membership to the control group) or individual levels of multi-level factors (e.g. one of, say, three possible cell types).

We use the logit link function $\pi_{i}=\exp\left(\overset{t}{\underset{j=1}{\sum }}X_{i,j}\eta_{j}\right)/\left(1+\exp\left(\overset{t}{\underset{j=1}{\sum }}X_{i,j}\eta_{j}\right)\right)$, so that an increase of the regression parameter *η*_*j*_ by *b* units can be interpreted as the log odds ratio $b=\log\left(\left(\pi_{i}^{\prime }/\left(1-\pi_{i}^{\prime }\right)\right)/\left(\pi_{i}/\left(1-\pi_{i}\right)\right)\right)$, where *π*_*i*_ is the mean methylation level corresponding to the parameter vector *η* and *π*^′^ is its value when *η*_*j*_ is increased by *b* units.

### Fitting

The beta-binomial regression is fit separately for each target site. Given the model matrix *X* and the read proportions (*m*_1_,*n*_1_),…,(*m*_*s*_,*n*_*s*_), indicating the methylation status of the target site across *s* samples, the values of the regression parameter vector *η* and distribution parameters *γ*, *π*_*i*_s are estimated using the method of maximum likelihood. To determine if a site is differentially methylated with respect to the test factor, we fit two regression models: the full model and the reduced model without the test factor. The significance of differential methylation is determined by comparing the full and the reduced models using the log-likelihood ratio test.

The p-values for the individual sites from the log-likelihood ratio test are combined together to increase the power to detect differential methylation. This approach enables the detection of differential methylation even in loci that have low coverage in all samples.

### Combining evidence for differential methylation

The p-values are transformed using weighted Z test (also known as Stouffer-Liptak test), employing an approach proposed by Kechris and others [23]. Briefly, the idea is to use Z test to combine the p-value associated with the target site with the p-values of its neighbors. A sequence of p-values *p*_1_,…,*p*_*n*_ corresponding to proximal sites is first transformed to a sequence of Z-scores *z*_*i*_=*Φ*^−1^(1−*p*_*i*_), for *i*=1,…,*n* and then combined using

$$p_{z}=1-\Phi \left(\frac{\underset{i}{\sum }z_{i}}{\sqrt{n+\underset{i<j}{\sum }\text{cor}\left(z_{i},z_{j}\right)}}\right)$$

as described, for instance, by Zaykin [24]. The correlation coefficients are calculated as previously described [25]. (The appropriate value of the parameter defining the width of the window in which to calculate the correlation between the p-values and subsequently combine them is discussed in the Additional file 1).

### Implementation

The DM detection method described above is implemented in RADMeth: Regression Analysis of Differential Methylation software available at http://smithlabresearch.org. The website contains the source code released under GNU GPL open-source license, binaries for Linux & Mac, and a manual.

## Results and discussion

### Simulated data

#### Comparison of DM detection methods

To compare performance of DM detection methods we simulated a dataset containing 12 samples (6 cases and 6 controls). Each sample consisted of CpG read proportions with coverages taken from a recent study of the mouse brain [26]. The number of reads indicating methylation of each CpG in each sample were taken from the binomial distribution. The distributions of the probability of success parameter (i.e. methylation level) of the binomials were taken from the work of Rakyan and others [7]: The non-differentially methylated CpGs in all samples had intermediate methylation levels taken from Beta (2,2), while differentially methylated CpGs in control and case samples had low methylation levels from Beta (1.5,6) and high methylation levels from Beta (6,1.5) respectively.

To get a sense for changes in false positive rates caused by variation in dispersion, we constructed two additional datasets. One of the datasets was obtained from the original by changing the distribution of non-differentially methylated CpGs to Beta (1.5,1.5), corresponding to the increase in variance from 1/20 to 1/16. The second dataset had no dispersion in the methylation levels of non-differentially methylated CpGs: all of them were set to 0.7. Each dataset contained 54,449 DM CpGs lying in 1,000 randomly selected regions that were required to have between 10 and 100 CpGs with no more than 200 bp between each pair of neighbors.

We evaluated the performance of each method by calculating the Jaccard index between the set of CpGs identified by the method as differentially methylated (which we denote by *M*) and the set of truly differentially methylated CpGs (denoted by *T*). The Jaccard index is given by |*M*∩*T*|/|*T*∪*M*|, i.e. the number of truly differentially methylated CpGs identified by a DM method divided by the total number of CpGs that are either truly differentially methylated or falsely identified as such. This way, the perfect method gives the Jaccard index of 1 and the worst possible method that misidentifies each differentially methylated CpG results in the Jaccard index of 0.

#### This is how we applied other existing methods

We recognize that the compared methods were each optimized for different contexts, and so our results do not in any way reflect their general value and validity. In running these other methods, we adjusted parameters so that each method would perform better on our tests, which could be significantly different from their intended contexts.

BSmooth is designed for estimating DM regions, so we identified all CpGs lying within DM regions as differentially methylated. To compute DM regions with BSmooth, we filtered out CpGs having coverage below 3 in more than 3 case samples or 3 control samples and used 4.5 t-statistics cutoff. Furthermore, we removed all DM regions with mean methylation difference below 0.1 and containing fewer than 3 CpGs.

Bisulfighter’s ComMet can be used to compare a pair of samples. So, to use it on our dataset we pooled read proportions from all case and all control samples. To decrease the rate of false positives, we discarded all CpGs with methylation difference below 0.6 between the pooled case sample and the pooled control sample.

When performing the analysis with DSS and MethylKit, we defined differentially methylated CpGs as those with q-value below 0.01. DSS had a low rate of false positives, but seemed to lack power to identify many differentially methylated CpGs. The false positive rate of MethylKit increased with variance. When using MOABS, we defined differentially methylated CpGs as those with credible methylation difference of 0.2 or above.

**Figure 1:**
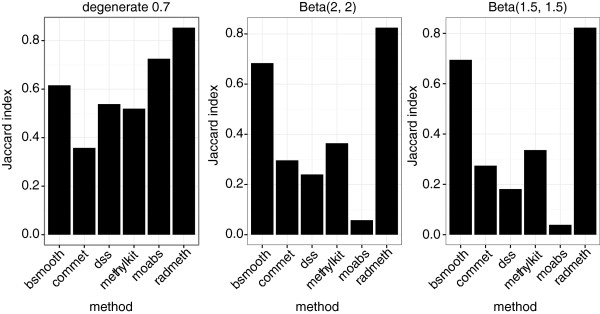
**Comparison of DM detection methods.** The Jaccard indexes comparing the truly differentially methylated CpGs to the CpGs identified as differentially methylated by each method. The panels are labeled according to the distributions of methylation levels of non-differntially methylated CpGs.

With RADMeth, CpGs with FDR corrected p-values below 0.01 were identified as differentially methylated. The correlation parameter was set to compute correlation between p-values of CpGs up to 200 bp from one another.The Jaccard indexes corresponding to each method applied to each dataset are described in Figure 1.

The DM detection method included in MethPipe methylation analysis pipleline is designed for detection of differential methylation within hypo-methylated regions and so is a less general DM detection method than the rest. To better highlight the differences between this method and ours, we made comparisons using an additional collection of datasets (see Additional file 1).

To check how well RADMeth performs on low-coverage data, we simulated another dataset consisting of 50 case and 50 control samples with the average coverage of 1.5 using same distributions of methylation levels as before (Beta (6,1.5) for cases, Beta (1.5,6) for controls, and Beta (2,2) for non-differentially methylated CpGs. The Jaccard index between the set of differentially methylated CpGs identified by RADMeth and true differentially methylated CpGs was 0.92.

### Applying RADMeth to real data

Our method was designed for large, multifactor WGBS datasets. It is inevitable that such datasets will be available in the public domain in the very near future, as on-going EWAS are completed. Analysis of these datasets requires accounting for (a) variation of methylation levels across replicates, (b) uncertainty associated with coverage, and also (c) adjustment for baseline differences due to population structure (e.g. age and sex of the involved individuals) or batch effects. Unfortunately, such datasets are largely absent from the public domain. Nevertheless, we chose two datasets – one multifactor and one involving a large number of samples – to illustrate our DM detection method. (See Additional file 1 for the description of parameters used to analyze each dataset).

#### A multifactor dataset

**Figure 2:**
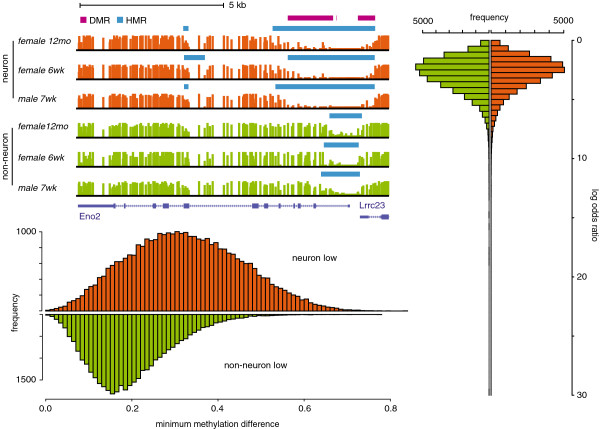
**DM regions between neuron and non-neuron samples.** (Top left) Methylation profile of the neuron specific enolase (Eno2) – a marker of neuron cells – across frontal cortex samples. (Right) Histogram of log-odds-ratios of DM regions containing at least 10 CpGs. (Bottom left) Histogram of minimum methylation differences of DM regions containing at least 10 CpGs.

We compared CpG methylation between neuron and non-neuron samples from mouse frontal cortex published in a recent study of methylation in the mammalian brain [26]. The 6 MethylC-Seq read libraries were processed with MethPipe [14] methylation analysis pipeline using standard parameter cutoffs. The resulting methylome samples had the mean coverage of 12.4 (s.d. 4.7). We computed DM CpGs and DM regions between neuron and non-neuron samples adjusting for baseline differences related to age and sex (12 month and 6 week old females, and 7 week old male). Top-left panel of Figure 2 contains a browser plot [27] with annotated DM regions and hypo methylated regions (HMRs) within a promoter of neuron specific enolase (Eno2), a well known marker of neuron cells [28,29]. The methylation profile of this gene across the frontal cortex samples reveals elongated HMRs upstream and downstream of the unmethylated promoter core in neuron samples compared to the ones in non-neuron samples, which constitute the DM regions.

Overall, there were about 72K DM regions containing 10 CpGs or more (see Figure 2 and also Additional file 1). Although predominantly glial, non-neuron samples consisted of multiple cell types. Hence the majority of DM regions, especially the ones corresponding to modest methylation changes, are likely to indicate difference between individual cell types and neurons. To obtain DM regions with consistent methylation changes between neurons and non-neurons in the majority of molecules comprising the samples, we selected DM regions with minimum methylation difference above 0.55. The 1,708 of these regions were lowly methylated in neurons and were associated with 1,089 genes. The GO term enrichment analysis, performed using DAVID [30], revealed a strong association of these genes with various aspects of neuronal development and function (see Additional file 2).

#### Large-scale dataset

The second dataset [31] consisted of 152 MethylC-seq libraries. The methylome samples obtained from these libraries with MethPipe [14] had mean coverage 11.2 (s.d. 2.7); 54 of these samples came from inflorescence (flower cluster) and the remaining 98 from the leaf of *Aradidopsis thaliana*. RADMeth identified 13,576 DM regions between the two groups of samples (see Additional file 1). Out of these, 5,049 DM regions containing at least 10 CpG sites were retained for downstream analysis.

**Figure 3:**
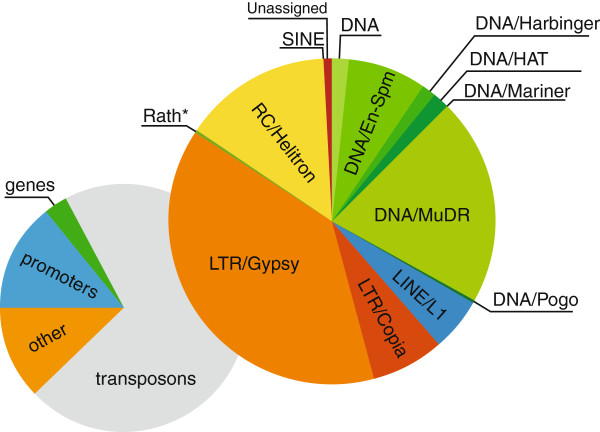
**Classification of *Arabidopsis *DM regions.** A summary of functional classification of 5,049 DM regions containing 10 CpG or more between 54 inflorescence and 98 leaf samples of *Arabidopsis thaliana*.

It is well known that methylation in *Aradidopsis* plays an important role in silencing of transposable elements (e.g. [32]), which are usually heavily methylated. Interestingly, most of the DM regions we found overlapped transposons (1.781 observed over expected ratio; see also Figure 3). The methylation differences between inflorescence and leaf samples were modest: above 0.1 for 1,271 DM regions and above 0.2 for just 129 regions, indicating relative loss of methylation within transposons in a relatively small fraction of sequenced molecules. Promoter and gene bound DM regions were underrepresented, with 0.19 and 0.28 observed over expected ratios respectively.

## Conclusions

In this work, we discussed the utility of the beta-binomial regression for comparing the distribution of read proportions corresponding to a single site (a single CpG site) across a set of WGBS samples under a given experimental design and then statistically combining the results of individual comparisons to estimate the DM status of individual sites and genomic regions.

Beta-binomial regression can model WGBS proportions in groups of samples corresponding to multiple experimental factors, including case-control experimental designs or more complicated designs involving, say, baseline adjustments for multiple cell types. In fact, simulations based on realistic distributions of methylation levels (see Additional file 1) show that, compared to the beta-binomial, the beta regression suffers loss of precision even at moderate coverage, while the extra-binomial variation increases the rate of false positives in the binomial regression. This suggests that beta-binomial regression is the appropriate way to analyze WGBS data in multifactor experiments.

The power to detect methylation changes of a given magnitude (e.g. log-odds ratio) varies with coverage. Because the coverage at the genome-wide scale is typically very uneven, it is possible to detect small methylation changes in regions with high coverage and only much larger methlation changes when the coverage is low. Combining the p-values associated with proximal cytosines boosts the significance of sites residing within DM regions making the detection of even lowly covered regions possible. There are other methods besides the Z test for combining the p-values, with Fisher’s method being the most well known. As explained by Rice [33], Fisher’s method is best suited for testing the existence of at least one significant test among the ones being combined, while the Z test is more appropriate in situations requiring the consensus among all of the combined tests, suggesting that the Z test is more appropriate for our purposes.

We note that, in agreement with earlier work [15], when dealing with the experiments involving small number of samples, there maybe little choice but to increase the power to detect differential methylation by leveraging methylation status of multiple sites either locally, as it is done by smoothing- and HMM-based methods or genome-wide, as done by some methods based on beta-binomial distribution.

In summary, the DM detection method described in this work is capable of (1) accurately modeling the uncertainty associated with coverage, (2) account for the extra-binomial variation that can arise from multiple biological and technical sources, (3) detect DM sites and regions even in loci having low coverage across all available samples, and (4) do all this while adjusting for the baseline differences corresponding to the relevant experimental factors.

## Competing interests

The authors declare that they have no competing interests.

## Authors’ contributions

ED and ADS made contributions to every component of this work. Both authors read and approved the final manuscript.

## Supplementary Material

Additional file 1 Comparison of beta, beta-binomial, and binomial regressions. Click here for file

Additional file 2 **GO term enrichment of genes associated with DM regions lowly methylated in neuron samples (performed using DAVID [**[30]**]).** Click here for file
