# Supplementary material for: Using beta-binomial regression for high-precision differential methylation analysis in multifactor whole-genome bisulfite sequencing experiments
Source: BMC Bioinformatics. 2014 Jun 24;15:215. doi: 10.1186/1471-2105-15-215 (PMC4230021; doi:10.1186/1471-2105-15-215)
Supplement: Additional file 1 — Comparison of beta, beta-binomial, and binomial regressions. [file 1471-2105-15-215-S1.pdf]

Additional Information

Comparison of beta, beta-binomial, and binomial regressions

We have compared the ability of beta, beta-binomial, and binomial regressions to detect differential methylation of individual sites on three simulated case/control datasets. All datasets involve 60 samples (30 cases and 30 controls) with the distributions of methylation levels in each group as described by Rakyan and others [2]. The coverage in each sample was set to 25. The receiver operating characteristic (ROC) curves corresponding to the first dataset are depicted on the left panel of Figure 1. These curves indicate that beta-regression performs considerably worse than beta-binomial when the observed methylation levels are estimated by the count ratios. The binomial regression is somewhat more sensitive than beta and beta-binomial regressions, but is incapable of achieving low false positive rates on this dataset. The situation with the other two datasets is similar (Figure 1 center, right).

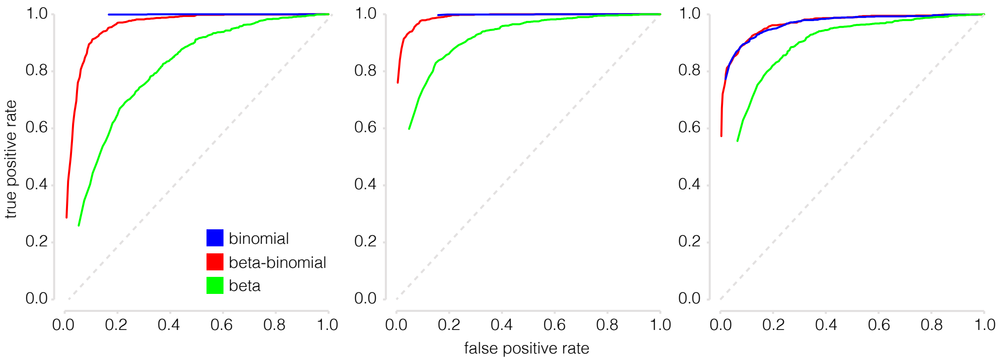

Figure 1: ROC curves (Left) The methylation levels of the control samples are Beta$(1.5, 6)$ distributed while the cases are a mixture with the methylation level having Beta$(1.5, 6)$ distribution in 36% of samples and Beta$(6, 1.5)$ distribution in 64% of samples. (Center) The methylation levels of controls are Beta$(1.5, 6)$ distributed and the levels of cases are a mixture of Beta$(2, 2)$ (in 72% of samples) and Beta$(6, 1.5)$ (in 28% of samples). (Right) The methylation levels of cases and controls have Beta$(36, 4)$ and Beta$(38, 2)$ distributions respectively.

dataset	DM CpGs low	DM CpGs high	Non DM CpGs	coverage
A	Beta(1.5, 6.0)	Beta(6.0, 1.5)	Beta(2.0, 2.0)	15
B	Beta(2.0, 2.0)	Beta(6.0, 1.5)	Beta(2.0, 2.0)	15
C	Beta(3.0, 4.5)	Beta(6.0, 1.5)	Beta(2.0, 2.0)	20
D	Beta(2.0, 2.0)	Beta(6.0, 1.5)	Beta(2.0, 2.0)	25

Table 1: The description of the datasets on which RADMeth was compared with MethPipe's DM-detection method. The columns give the dataset id, the distribution of methylation levels of DM CpGs in samples where they had low and high methylation levels, the distribution of methylation levels of non-differentially methylated CpGs, and the average coverage. Each dataset contained 12 samples (6 with low methylation at DM CpGs and 6 with high methylation).

RADMeth and MethPipe's DM detection method

The DM detection method included in MethPipe methylation analysis pipeline is designed to detect differential methylation within hypo-methylated regions (i.e. regions with consistently low methylation) and so it is a less general DM detection method than the other DM-detection methods described in this work. We compared this method to RADMeth on four datasets. The data sets differed from each other by the mean methylation levels of DM CpGs in one group of samples (see Table 1): It was 0.2, 0.5, 0.4, and 0.5 for the datasets (A), (B), (C), and (D) respectively. The coverage of the datasets has also varied, but did not have a significant impact on the results.

Because MethPipe's DM-detection method was designed to compare only two samples at a time, we combined the replicates by pooling. The Jaccard index between the set of CpGs identified as differentially methylated by this method and the set of true differentially methyalted CpGs was 0.87 for the dataset (A), 0.33 for the dataset (C), and 0 for the data sets (B) and (D). The method performed well on the data set (A) because the vast majority of DM CpGs resided in hypo-methylated regions due to their low methylation levels. With the increase of the mean methylation levels, the performance of this method drops very quickly. The RADMeth-based analysis yielded the Jaccard indexes exceeding 0.80 for all datasets.

	F6	M7	F12	neuron
NeuronFemale12Mo	0	0	1	1
NeuronFemale6Wk	1	0	0	1
NeuronMale7Wk	0	1	0	1
NonNeuronFemale12Mo	0	0	1	0
NonNeuronFemale6Wk	1	0	0	0
NonNeuronMale7Wk	0	1	0	0

Figure 2: The model matrix describing the mouse frontal cortex dataset. The first three columns of the matrix mark samples taken from individuals of the same age and sex. The last column marks neuron samples and corresponds to the test factor.

DM regions in *Arabidopsis* [3] and mouse cortex datasets [1]

To analyze each dataset we used appropriate model matrices (depicted on Figures 2 and 3) and combined the p-values corresponding to each CpG site with the p-values of CpG sites located within the 200 bp from it (the `-b` parameter in the `wand` regression module, see RADMeth manual for more information).

RADMeth identified 5K DM regions spanning at least 10 CpG sites in the Arabidopsis dataset and 72K DM regions spanning at least 10 CpGs in the mouse cortex dataset. The histograms with lengths, numbers of CpG sites, and GC content of these regions are depicted on Figure 4.

Two-group comparisons: replicates and coverage

The ability of RADMeth and the other methods to detect differential methylation depends on the number of replicates, their coverage, and the design of the experiment. We performed a simulation study to determine the minimum coverage required to reliably detect differential methylation in two-group datasets containing 3, 4, and 5 replicates in each group (using the parameters for dataset (A) described in Table 1 which correspond to rather large methylation changes between the groups). The 3 replicate case required the minimum average coverage of 9 to detect differentially methylated regions giving the Jaccard index of 0.75 or above. In the 4 and 5 replicate case, the datasets with average coverage of 7 and 4 gave Jaccard indexes above 0.80.

	int	leaf
leaf_rep_1	1	1
leaf_rep_2	1	1
...		
leaf_rep_54	1	1
infl_rep_1	1	0
infl_rep_2	1	0
...		
infl_rep_98	1	0

Figure 3: The model matrix describing the *Arabidopsis* dataset. This model matrix encodes a simple two-group experimental design. The last column corresponds to the test factor.

Combining evidence for differential methylation

We recommend calculating correlation and subsequently combining the p-values of sites located within 200 bp. In our experience correlation typically becomes much weaker beyond this point, so the users do not generally need to alter this parameter. However, it may be approprtiate to increase the value of this parameter when analyzing very noisy data.

References

[1] Ryan Lister, Eran A Mukamel, Joseph R Nery, Mark Urich, Clare A Puddifoot, Nicholas D Johnson, Jacinta Lucero, Yun Huang, Andrew J Dwork, Matthew D Schultz, et al. Global epigenomic reconfiguration during mammalian brain development. *Science*, 341(6146), 2013.

[2] Vardhman K Rakyan, Thomas A Down, David J Balding, and Stephan Beck. Epigenome-wide association studies for common human diseases. *Nature Reviews Genetics*, 12(8):529–541, 2011.

[3] Robert J Schmitz, Matthew D Schultz, Mark A Urich, Joseph R Nery, Mattia Pelizzola, Ondrej Libiger, Andrew Alix, Richard B McCosh, Huaming Chen, Nicholas J Schork, et al. Patterns of population epigenomic diversity. *Nature*, 2013.

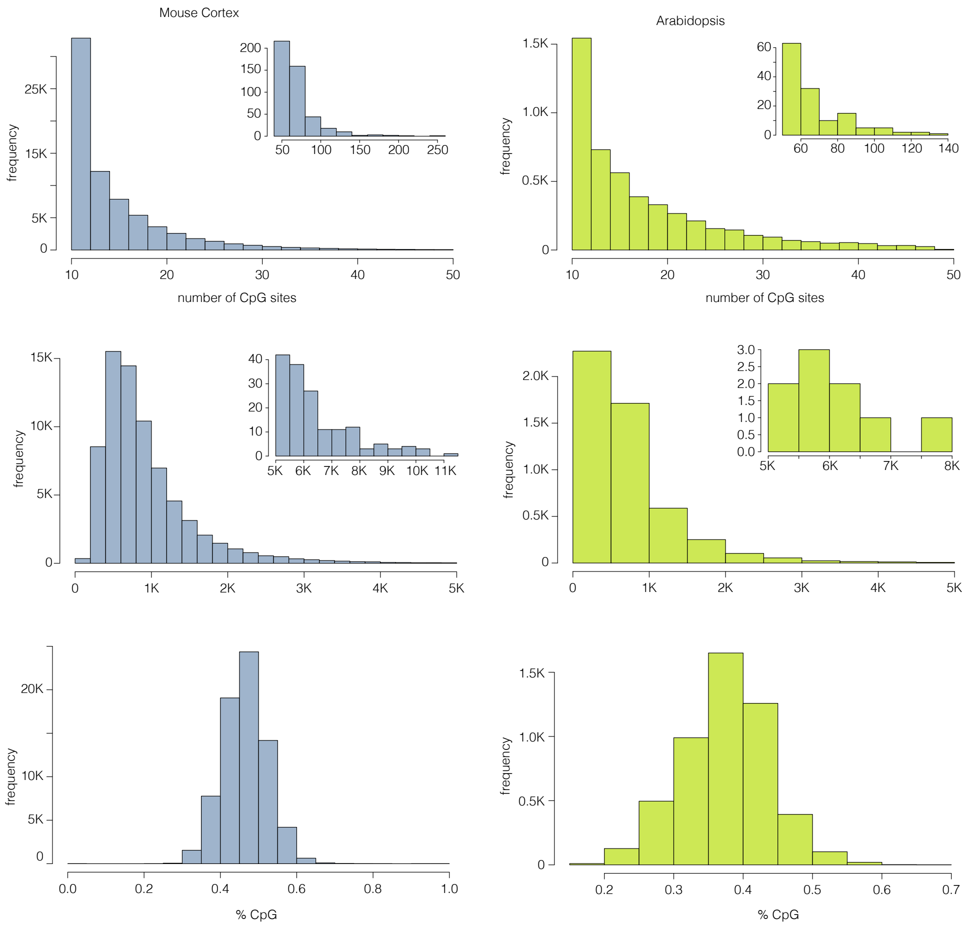

Figure 4: Histograms of the number of CpG sites (top), length (middle), and GC content (bottom) of the DM regions from the mouse frontal cortex (left) and *Arabidopsis* (right) datasets.
